# Patterns and drivers of Human Visceral Leishmaniasis in Pernambuco (Brazil) from 2007 to 2018

**DOI:** 10.1371/journal.pntd.0011108

**Published:** 2023-02-08

**Authors:** Carolina Angélica Libório Machado, Denis Valle, Mauricio Claudio Horta, Ana Yoko Ykeuti Meiga, Anaiá da Paixão Sevá

**Affiliations:** 1 Universidade Federal do Vale do São Francisco, Petrolina. Pernambuco, Brazil; 2 School of Forest, Fisheries, and Geomatics Sciences, University of Florida, Gainesville, Florida, United States of America; 3 School of Natural Resources and Environment, University of Florida, Gainesville, Florida, United States of America; 4 Universidade Estadual de Santa Cruz, Ilhéus, Bahia, Brazil; National Institute of Allergy and Infectious Diseases, UNITED STATES

## Abstract

Visceral leishmaniasis (VL) is the second most common protozoosis that affects people around the world. The aim of this study is to understand how environmental and socioeconomic factors, as well as VL control and surveillance interventions, influence the spread and detection of VL cases in Pernambuco state (Brazil). A novel model was developed to analyze cases of VL between 2007 and 2018, enabling the quantification of the association of these variables with two processes: the probability of “invasion” (emergence of new cases) at municipalities by VL, and the probability of detecting cases not reported in municipalities that have already been invaded. Pernambuco state identified 1,410 cases of VL between 2007 and 2018, with an average of 128 cases per year and average incidence of 1.28/100 thousand people. These cases were distributed in 77.1% (142/184) of the municipalities, and 54.8% (773/1,410) of them were autochthonous. Our model reveals that the proportion of agriculture was positively associated with VL invasion probability. We also find that municipalities that are closer to notification centers and/or that have received technical training and support tend to have higher detection rates of VL cases. Taken together, these results suggest that a municipality with almost no agriculture and that received technical training, located close to a notification center, is unlikely to be invaded if no cases have ever been detected. On the other hand, a municipality that is far from the notification center, with no technical training, with a large agricultural area might have already been invaded but the surveillance system might have routinely failed to detect VL cases due to low detection probability. By disentangling the processes of invasion and detection, we were able to generate insights that are likely to be useful for the strategic allocation of VL prevention and control interventions.

## Author summary

Visceral leishmaniasis (VL) is an important neglected zoonosis around the world. We evaluated how environmental and socioeconomic factors, as well as VL control and surveillance interventions, influence the spread and detection of VL cases in Pernambuco state (Brazil), which is endemic. Our results reveal that municipalities with almost no agriculture that received technical training and are located close to a notification center are unlikely to have VL infected individuals if no cases have ever been detected. On the other hand, a municipality distant from the notification center, with no technical training, and with a large agricultural area might have already had unreported cases. These results can serve as a foundation to improve the personnel training and notification system strategies adopted by VL Public Health Programs.

## 1. Introduction

Visceral leishmaniasis (VL) is the second most common protozoosis that affects people around the world [1] and is a major public health problem in five regions: Americas, East Africa, North Africa, West Asia and South-East Asia. According to the World Health Organization, in 2018, 83 countries were endemic or had reported cases of VL and currently more than one billion people live in VL endemic areas with the risk of infection estimated at 30,000 new cases per year [2].

In the New World, this zoonosis is caused by *Leishmania infantum* sin. *L*. *chagasi* and is transmitted by sand flies from the genus *Lutzomyia* [3]. In Brazil, several strategies are adopted by the VL Prevention and Control Program (VLPCP), focused on the vectors (e.g., monitoring and reduction of sand fly populations), intermediate hosts (e.g., surveillance of cases and elimination of infected reservoirs, such as domestic dogs), and humans (early diagnosis and treatment of human cases and health education) [4]. Importantly, notification of suspected or confirmed cases is legally mandatory [5] but unfortunately, despite the strategies implemented by the VLPCP, the number of VL cases and its geographical dispersion continues to increase throughout the territory [1].

Between 2001 and 2017, VL cases in Brazil have been distributed mainly in the northeast, southeast, and midwest regions of the country [6], with the northeast region having the highest number of cases of VL [7,8]. Favorable climate (e.g., low altitude and high diurnal range in temperature; [9]), proximity to major infrastructure projects (e.g., gas pipelines and highways; [10]), and deforestation [11] have been shown to be important determinants of VL in endemic areas in the country. In the northeast region of Brazil, in particular, VL has been shown to be more prevalent in the Cerrado and Amazonian biomes [8]. Similarly, a comparison of several ecosystems in the North region of Brazil revealed that there was higher prevalence of VL in domestic dogs in the Amazonian biome [12].

Socioeconomic factors are also important drivers of this disease. For example, poverty has been found to be strongly associated with the disease in Brazil and across the world [9,13,14] and increased risk of developing leishmaniasis has been linked to poor housing conditions, lack of sanitation, and restricted access to health care. Importantly, while leishmaniasis in general affects mainly developing countries and remote places, such as rural villages [15], it has been noted that this disease is increasingly more common in urban rather than rural areas in Brazil [12,16].

Relatively few articles have examined temporal changes on the spatial dispersion of VL in Brazil. A spatial analysis in the southeast of the country found that the expansion route and dissemination of VL has been from west to east of the state of Mato Grosso do Sul, eventually reaching São Paulo State. This dispersion was related with the construction of a federal highway, a railroad, and the Bolivia-Brazil gas pipeline, all of which are associated with intense human traffic along them as well as significant environmental disturbance [17]. In São Paulo state, vector dispersion was found to be influenced by proximity to the same gas pipeline and high temperatures whereas rainfall influenced human VL cases [10]. Finally, in Minas Gerais state, Da Silva et al. [18] observed that six mesoregions were responsible for the expansion and maintenance of VL, with only two of these mesoregions showing a large increase in incidence rates. Critically, despite the fact that the disease is spreading in the country, there has been comparatively little research on the drivers of VL dispersion, particularly for the most endemic northeast region.

An important challenge in understanding the drivers of geographical dispersion of VL is under-reporting. In Brazil, it is common to have inconsistencies in reported cases [8] and underreporting due to a wide range of problems, including delay in seeking health care, delay or failure of diagnosing the disease, and/or failure in the notification process (e.g., due to the complexity of the notification protocol and reporting system, and/or a lack of appreciation of the importance of notification). Importantly, because VL cases are strongly associated with low education and socioeconomic levels [8,19], underreporting is believed to be widespread despite mandatory notification. In this study, we assess the role of potential drivers of dispersion and detection of VL cases in Pernambuco state, a state located in the Northeast region of Brazil, between 2007 and 2018, through the use of spatial descriptive analyses and a novel statistical model that accounts for underreporting.

## 2. Methods

### 2.1. Study area

**Fig 1:**
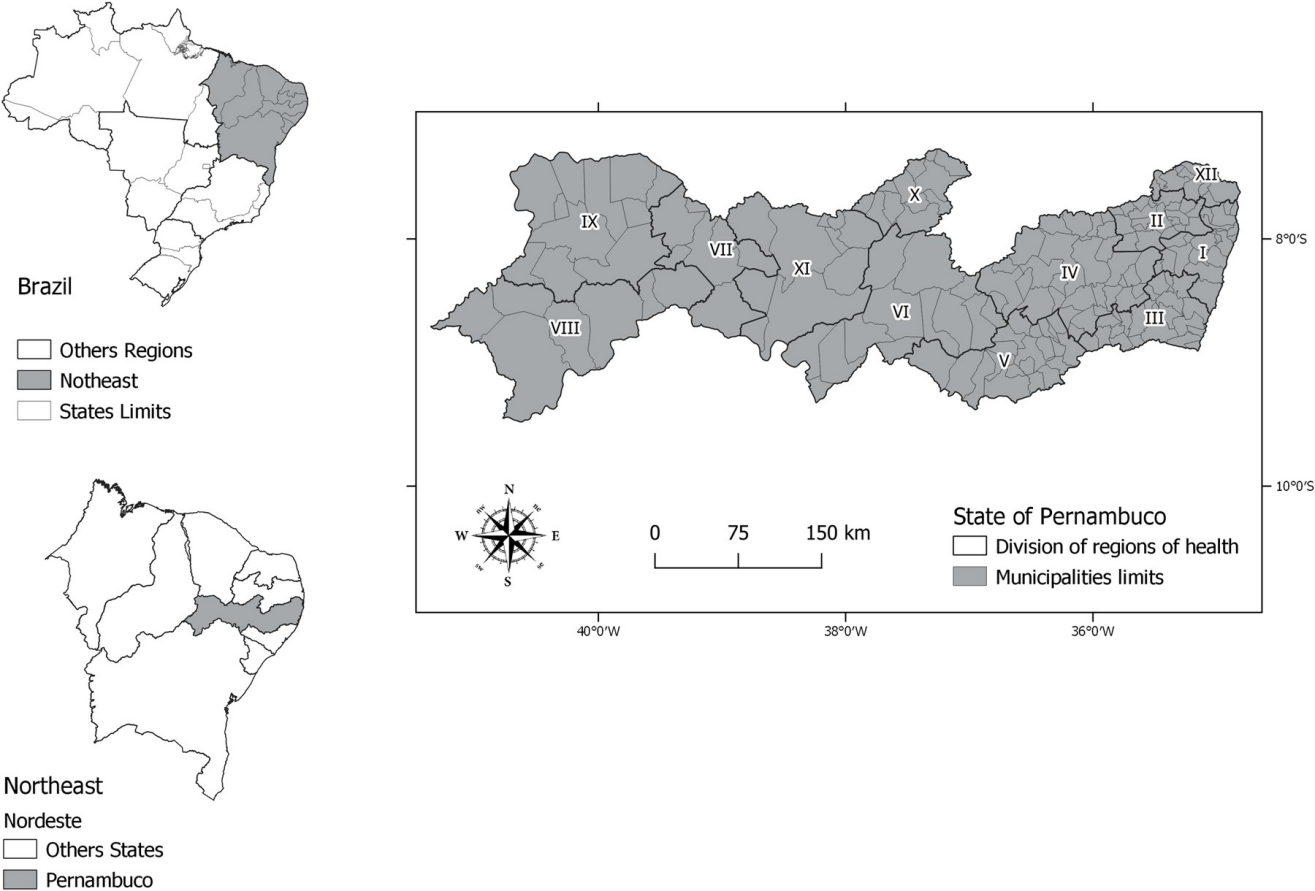
State of Pernambuco in the northeast region of Brazil, and its Health Management Regions, shown with Roman numerals. Legend. Base layer of map: https://www.ibge.gov.br/geociencias/organizacao-do-territorio/malhas-territoriais/15774-malhas.html?=&t=acesso-ao-produto.

Pernambuco is one of the 27 Brazilian states and is located in the northeast region of the country. It has 185 municipalities grouped in 12 Health Management Regions (HMR) (Fig 1). However, for our analysis, we excluded the municipality of Fernando de Noronha because it is an island far from the continent (545 km away), having very different environmental, economic, and health system characteristics. Finally, the population of the state is estimated as exceeding 9 million, and the Human Development Index (HDI) is considered medium (0.673).

### 2.2. Data on VL incidence

All data on VL cases from 2007 to 2018 were obtained from the Coordination of Zoonosis at the Health Surveillance Secretariat of the State of Pernambuco. These data consist of information for each VL disease case, including municipality, year, age group, gender, onset of symptoms, outcome of the case, and municipalities of residence, health assistance, and notification. Since these data do not reveal the identity of the subject and are made publicly available (upon request) by the Health Surveillance Secretariat, these data do not require authorization to use and do not require registration/evaluation by the Brazilian Ethics Committee [20]. The incidence of VL was calculated as cases divided by the estimated population for each municipality, where the estimated population for each municipality was provided by the Brazilian Institute of Geography and Statistics and obtained from the data system of the Ministry of Health [21].

### 2.3. Characterization of notification routes

To help characterize which municipalities are likely to have difficulty reporting their cases, we calculated the Euclidean distance between the municipality centroid where each case was notified and the municipality centroid of the origin of the cases (assumed to be the patient’s residence).

### 2.4. Data for statistical model

A statistical model was developed to represent two processes: emergence of cases in municipalities that have never had any VL cases before (i.e., VL “invasion”), and detection of cases in municipalities that have already been invaded (i.e., VL “detection”). Taken together, this model enables us to distinguish between municipalities without any cases versus municipalities with cases that have not been reported due to poor surveillance. Furthermore, this model enabled us to assess the effect of socioeconomic factors, environmental factors, and interventions by the state health agency on each of these processes.

Aside from data on VL incidence, information related to VL surveillance and control interventions were also included, describing when and which municipalities had been provided with technical training and support, as well as when and which municipalities were prioritized by the disease control program (SANAR program). We also obtained the number of health facilities and the sanitation index for each municipality from the DATASUS system. The sanitation index was calculated as the proportion of people in houses that dispose of their sewer without treatment (e.g., by relying on septic tanks, rudimentary cesspool, or simply disposing of their sewer on ditches, rivers, lakes, or sea).

Annual land use and land cover (LULC) data were obtained from Mapbiomas [22]. The total area of each LULC class for each municipality was extracted, and after dividing by total area of the municipality, resulted in the percentage of each LULC class in each municipality. This data manipulation was performed by using the shapefile of municipality, state and countries limits (available in the Brazilian Institute of Geography and Statistics website) limits and the *raster* package in R software (version 3.6). Maps were created, and spatial analyses were performed, using QGIS (version 2.8).

#### Variables for invasion risk

Previous studies of VL have suggested that proximity to forests and presence of livestock may increase the abundance of the main vector *Luztomyia longipalpis* [23–25]. For this reason, we decided to focus our analysis on the LULC classes “Mosaic of Agriculture and Pasture” (pasture and agricultural areas with features indistinguishable from pasture) and “Forest Formation” (dense, open, and mixed forests). We did not include climatic variables (e.g., mean temperature and precipitation) because a preliminary analysis revealed that these variables were strongly correlated with the “Forest Formation” variable. Finally, given that VL is often associated with poor housing conditions and lack of environmental sanitation [13], we also included sanitation index as a potential risk factor for VL invasion.

#### Variables for detection probability

We were also interested in determining how several variables influenced the probability of VL detection, including technical support, technical training of health professionals, distance to notification centers, and number of health facilities available for care and notification of the population. Notification centers are defined by the State Health Department and are the center of each Health Management Region. We calculated the distance between these notification centers and the origin of the cases (considering the centroid of the referred municipality). We hypothesize that municipalities that are more distant from these centers are likely to have more difficulty in identifying and reporting their cases, resulting in lower detection probability. Furthermore, we hypothesize that municipalities that received technical support and training for health professionals throughout the year are likely to have higher detection probabilities.

### 2.5. Description of statistical model

We rely on a statistical model that has a similar structure to that of occupancy models, a class of models that is frequently used for wildlife studies. These models attempt to understand both the factors that determine occupancy by the target species and the factors that influence its detection. The application of this model to the present study enables us to determine which factors influence the invasion probability of municipalities, and which factors influence the detectability of new VL cases.

Let *y*_*it*_ denote if cases were observed in municipality *i* at time *t* (0 and 1 represent the absence and presence of cases, respectively). We assume the following *Bernoulli* distribution for this variable:

$$y_{it}∼Bernoulli(z_{it}p_{it})$$

where *z*_*it*_ is the true invasion status and *p*_*it*_ is the detection probability of municipality *i* at time *t*. We assume that the detection probability depends on several covariates through the following logistic relationship:

$$p_{it}=\frac{exp(x_{it}{}^{T}\beta )}{1+exp(x_{it}{}^{T}\beta )}$$

where ***x***_***it***_ is the design vector containing a leading 1 for the intercept and the covariates potentially associated with detection probability and ***β*** is a vector of regression coefficients. The regression coefficients within ***β*** quantify if and how each covariate influences detection probability.

In relation to the partially latent true invasion statuses, we rely on a logistic regression structure to model the invasion process. More specifically, we assume that the probability of invasion for municipalities that have not been invaded yet is given by:

$$p(z_{it}=1\left|z_{i(t-1)}=0\right.)=\frac{exp(w_{it}{}^{T}\alpha )}{1+exp(w_{it}{}^{T}\alpha )}$$

where ***w***_***it***_ is the design vector containing a leading 1 for the intercept and the covariates potentially associated with the invasion process and ***α*** is a vector with the corresponding coefficients. In this expression, the regression coefficients within ***α*** determine if the corresponding covariate is associated with invasion probability and if it is a risk or protective factor. On the other hand, a municipality that has already been invaded is assumed to remain invaded through the subsequent years even if no additional cases were subsequently detected:

$$p(z_{it}=1\left|z_{i(t-1)}=1\right.)=1$$

Finally, for the first time step (i.e., *z*_*i*1_), we assumed that these latent statuses are given by:

$$z_{i1}∼Bernoulli(\gamma )$$

where 0 < γ < 1. We finish specifying this model by assuming the following prior distributions:

$$\beta ∼N(0,T_{\beta })$$

$$\alpha ∼N(0,T_{\alpha })$$

γ∼Unif(0,1)

where ***T***_***β***_ and ***T***_***α***_ are diagonal matrices specified by the modeler. These diagonal matrices contain the prior variances of the regression coefficients.

## 3. Results

### 3.1. Descriptive statistics

**Fig 2:**
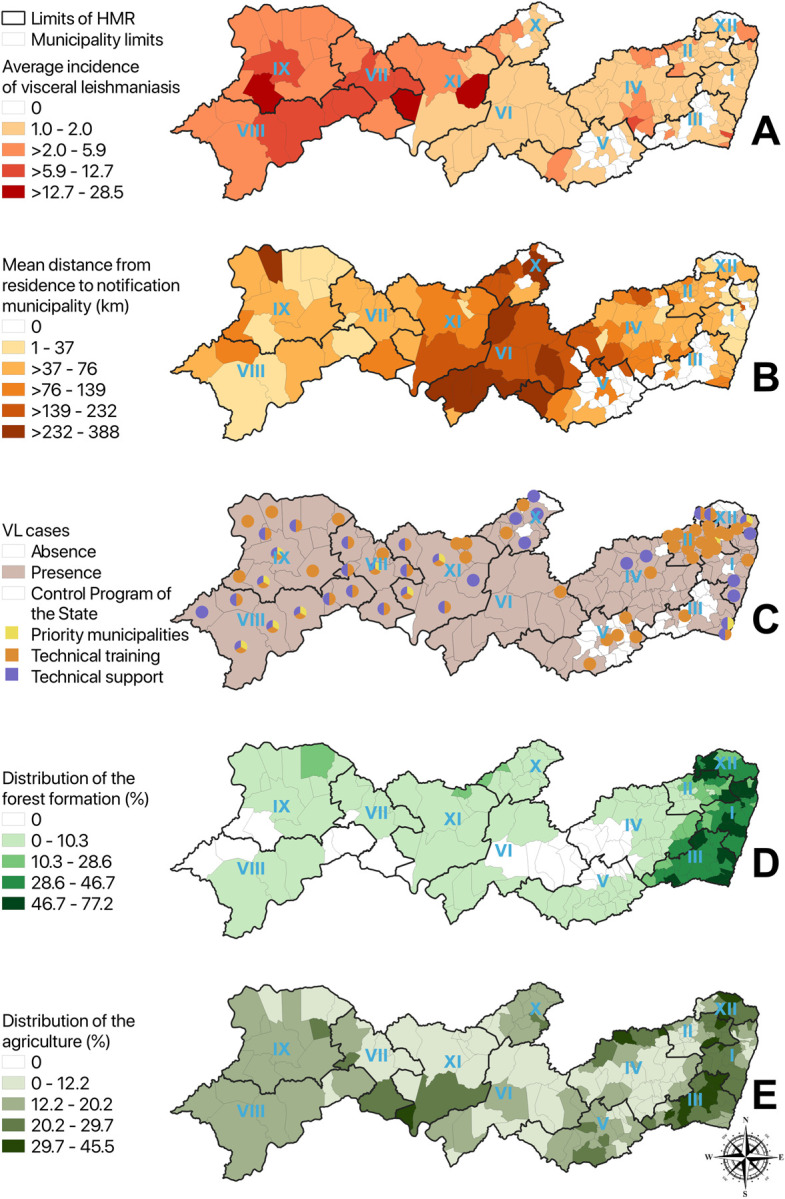
Spatial distribution of VL incidence (per 100 thousand habitants), mean distance from residence to notification municipalities, VL prevention and control interventions, and environmental risk factors (forest and agricultural areas). Roman numbers correspond to the Health Management Regions. Legend. Base layer of map: https://www.ibge.gov.br/geociencias/organizacao-do-territorio/malhas-territoriais/15774-malhas.html?=&t=acesso-ao-produto.

Pernambuco state notified 1,410 cases of VL between 2007 and 2018, with an average incidence of 1.28 per 100 thousand habitants. Approximately half of all cases (54.8%; 773/1,410) were autochthonous cases, 33.1% (467/1,410) were considered of undetermined infection location, and 11.9% (168/1,410) were not autochthonous. Based on the residence location provided by the patient, we find that all cases occurred in 76.6% (141/184) of the municipalities (Fig 2A). Importantly, 23.4% (43/184) of the municipalities did not have cases, despite the fact that almost all of them were bordered by municipalities that have cases. These results suggest that municipalities that never had any case probably have already been invaded but their cases might not yet have been diagnosed or notified.

The average distance from the municipality of origin of the cases to the municipality of notification within the state of Pernambuco (Fig 2B) was 48.42 km (median equal to 39.98 km). However, many cases were reported in Recife (capital of the state), requiring patients to travel a mean distance of 120 km. Importantly, for many municipalities, distances traveled were even higher, varying from 301 km (Custodia in HMR VI) to 425 km (Floresta in HMR XI).

Municipalities that have been provided with technical training and support, and priority municipalities for the VL control program within the state of Pernambuco, are shown in Fig 2C. There are 11 priority municipalities which were deemed to have a risk of moderate to intense transmission with the occurrence of deaths. All of these priority municipalities also received technical support. Interestingly, it is possible to observe that most of the priority municipalities, as well as the municipalities chosen to receive technical support and technical training, were in HMR VII, VIII, IX and XI, being located in regions defined as Sertão and São Francisco (in the western part of the state).

### 3.2. Statistical modeling results

**Fig 3:**
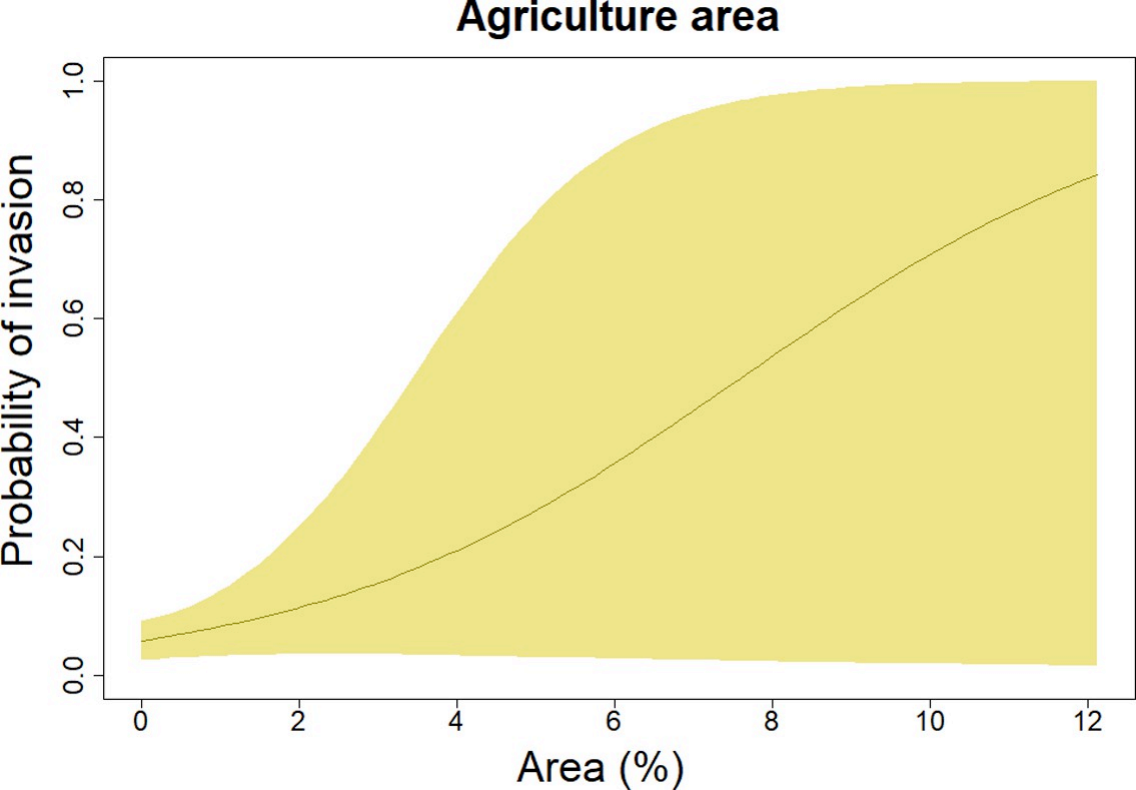
Estimated association between the percentage of the mosaic of agriculture and pasture area and probability of invasion of VL. The polygon represents pointwise 95% credible intervals whereas the line represents the posterior median.

The only variable that was statistically associated with VL invasion was the proportion of agricultural area but there was considerable uncertainty in this relationship (Fig 3). The distribution of these land covers in the Pernambuco state can be seen in Fig 2D and 2E. While there was some evidence of a negative relationship between sanitation and VL invasion, this association was not statistically discernible. Finally, we did not find evidence of an association between invasion probability and forest cover (Table 1).

**Table 1:** Estimated effect of covariates on probability of invasion of VL.

Covariate	Median	95% CI		min[p(α<0), p(α>0)]
Sanitation index	-0.54	-1.63	0.34	0.130
Mosaic of agriculture and pasture	1.03	-0.27	2.31	0.041
Forest formation	0.12	-0.86	0.73	0.376

**Fig 4:**
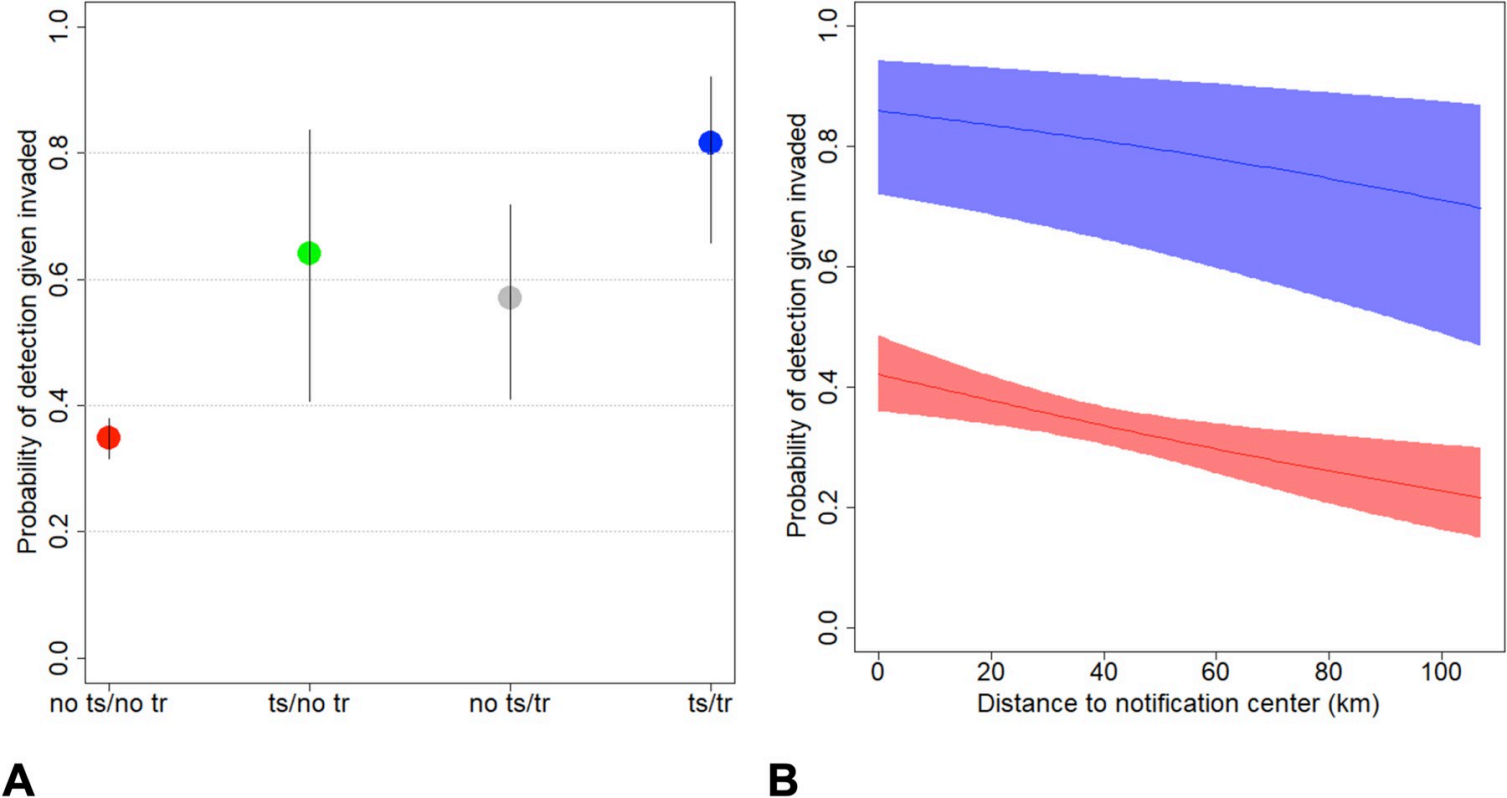
Relationship between detection probability for invaded municipalities and different covariates. Panel A displays the estimated relationship between detection probability and municipalities receiving technical support (“ts”) and/or technical training (“tr”). Points represent the posterior median and vertical lines are the corresponding 95% credible intervals. Panel B shows the estimated relationship between detection probability and distance to notification center for municipalities that did not receive technical support or training (red polygon and line) and for municipalities that received both technical support and training (blue polygon and line). Polygons represent pointwise 95% credible intervals while lines are posterior medians.

We also found that municipalities with technical training and support tended to have higher detection probability (Table 2). The relationship between detection probability and technical support and training is illustrated in Fig 4A, showing how municipalities that received both technical support and training have a much larger detection probability compared to those that only received one of these interventions or that received no intervention at all.

**Table 2:** Ability of detection of VL cases, and association with variables.

Covariate	Median	95% CI		min[p(β<0), p(β>0)]
Technical support	1.21	0.25	2.27	0.006
Technical training	0.91	0.26	1.57	0.003
Distance to notification center	-0.18	-0.31	-0.06	0.001
Number of health facilities	0.11	-0.04	0.25	0.079

Interestingly, our results also reveal that detection probability decreases as distance to the notification center increases (Table 2). The relationship between detection probability and distance to the notification center is shown in Fig 4B. Finally, the number of health facilities was not significantly associated with detection probability (min[p(β<0), p(β>0)] > 0.05).

**Fig 5:**
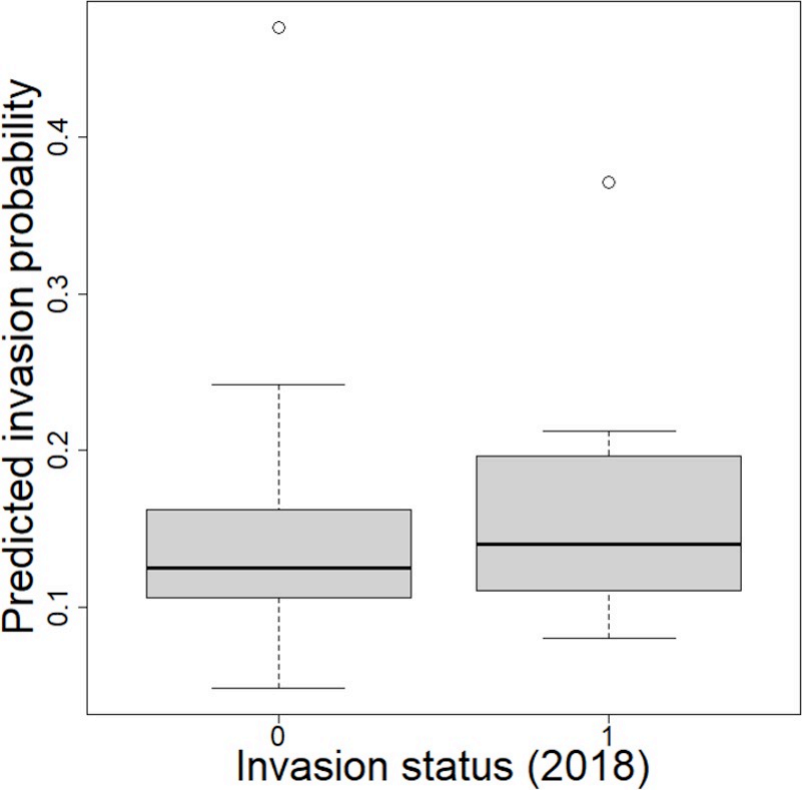
Comparison of the predicted invasion probabilities for the municipalities that had not been invaded by 2018 (invasion status = 0, n = 43) and for those that were invaded in 2018 (invasion status = 1, n = 7).

Using these results, the model was able to determine how many and which municipalities are likely to have VL cases, even if no cases have been reported there yet. For example, in the beginning of our time series (i.e., 2007), only 20% of the municipalities reported leishmaniasis cases but the model estimates this percentage as 52% (with a 95% credible interval of 40%–63%), suggesting that VL was already much more widespread than the reported case data suggested. Furthermore, although the model was trained on data from 2007 to 2017, this model can be validated by making predictions for 2018. This validation exercise reveals that, as expected, the proposed model tends to estimate higher invasion probabilities for the municipalities that were indeed observed to be invaded in 2018 (Fig 5). However, we also find that some of the municipalities not yet invaded by 2018 have very high (>40%) predicted invasion probabilities. It is possible that these municipalities have actually already been invaded but no cases have been reported yet. These results are based both on the environmental factors that determine the likelihood of invasion as well as the detection probabilities. For example, a municipality with favorable environmental factors but with low detection probability is likely to have already been invaded, even if no cases have been reported there. On the other hand, a municipality with less favorable environmental factors and high detection probability is unlikely to have already been invaded if no cases have been detected yet. A list of the 16 municipalities that did not have any VL case between 2007–2017 and that had the highest estimated probability of having already been invaded, is provided in Table 3.

**Table 3:** List of the sixteen municipalities that did not have any VL case between 2007–2017 and that had the highest estimated probability of having already been invaded.

Ranking	Municipality	HMR	Invasion Probability (%)
1	Itacuruba	XI	47.0
2	Tacaratu	XI	37.1
3	Camocim de São Félix	IV	24.2
4	Condado	XII	21.2
5	Venturosa	V	20.6
6	Frei Miguelinho	II	20.6
7	Ferreiros	XII	20.1
8	Itapetim	X	19.5
9	Ribeirão	III	19.3
10	Ipubi	IX	18.1
11	Correntes	V	18.0
12	Moreno	I	17.2
13	Itambé	XII	16.5
14	Ingazeira	X	16.2
15	Calçado	V	16.2
16	Chã Grande	I	16.2

## 4. Discussion

In this study, we have analyzed VL case data from 2007 to 2017 in Pernambuco using both descriptive statistics and a novel statistical model. Our descriptive analysis reveals that approximately half of the cases were autochthonous and approximately 1/4 of the municipalities still did not have cases, despite almost all of them being bordered by municipalities that have cases. We also found that most of the municipalities with largest distances between case origin and notification were located in Health Management Regions VI and X. Our statistical model revealed that detection probability tended to be lower for municipalities that were more distant to notification centers and that had not received technical support or training. Furthermore, this model revealed that the proportion of agricultural and pasture area was positively associated with invasion probability. These results, supported by the out-of-sample predictive exercise for 2018, enable us to differentiate the municipalities that are likely to already have been invaded from those municipalities that are likely to be free of undetected VL cases. We discuss below these findings and their implications in the context of what is currently known about VL and current VL policies.

### 4.1. Spatial distribution and geographical spread of VL cases

Our maps show a high concentration of VL cases in the HMR VII, VIII and IX, which corresponds to the geographic regions of São Francisco and part of the Sertão of Pernambuco state. This distribution of cases began to be observed in 1998 [26], corroborating the results from a previous study that found more cases in the west of the state and a smaller number of cases in the coast (far east) [8,26].

We found that municipalities that have a higher proportion of agriculture and pasture area are more likely to be invaded by VL in Pernambuco. Indeed, other studies have suggested that the presence of livestock may contribute to the proliferation of the main vector of VL, *Luztomyia longipalpis* [25], and this vector has been associated with degraded environments and more open vegetation such as Cerrado [27,28]. In Pernambuco in particular, the existing agriculture consists predominantly of sugar cane and, once sugar cane is harvested, there is substantial accumulation of organic matter in the field. Decomposing organic matter has been shown to be an important food source for the sandfly larvae [29] and its accumulation in the field can also attract rodents, an important blood source for the VL vector and a natural host of *L*. *infantum* [30,31].

Interestingly, we did not find a statistically discernible association of VL incidence with forest formation. It is important to note that, although natural forest formation is present throughout the state, this is not a homogeneous LULC class. More specifically, the extreme east (approximately 125 km from the coast) is composed by the Atlantic Rainforest biome (Fig 2D with higher percentage of forest), while the other ~550 km of extension to west is predominantly by composed by the Caatinga biome. The rainforest tends to be dense and humid while caatinga is drier and more open. It is possible that the combination of these two types of forests into a single LULC class hindered our ability to detect an association with VL risk. Indeed, previous research has revealed that *Lu*. *longipalpis* (the main VL vector species) does not require dense vegetation to survive [32] and it is more common for VL cases to occur in dry and hot areas of the northeast region of Brazil [8,28], such as the Caatinga biome. As with all analyses that rely on aggregate data, it is also possible that we failed to find associations with forest formation and sanitation index because of the spatial scale of our data. For example, it is possible that the relationship between some of these factors and vector abundance is present only at the micro-habitat scale, not at the municipality level.

By taking into account the detection process, our results suggest that approximately 51% of the municipalities had already been invaded in the beginning of the study period despite the fact that only 20% of the municipalities had reported VL cases in 2007.

### 4.2. Detection of VL cases

According to the State Health Department of Pernambuco, all municipalities have the capacity and autonomy to report their cases [33]. Although notification can be made in any health facility (e.g., from the basic health unit to the municipal epidemiological center), some municipalities have historically failed to notify their cases. This can be seen on Fig 3, where the municipalities located in the central region of the state (HMR VI and X) had greater mean distance to notification when compared to other regions. Critically, all cases from these municipalities were reported in the capital Recife (HMR I). This often occurs when the patient either directly seeks help in the best treatment center (located in the capital Recife) or is transferred to be treated there because he/she is in a serious condition, even if a diagnosis has not yet been made.

It is important to note that although 23.4% of the municipalities in Pernambuco did not report VL cases during the study period, this does not mean that these municipalities were disease free. Indeed, it is well known that symptomatic cases are only a small percentage among those infected [34]. Furthermore, aside from the failure in the diagnosis of the disease, failure in the notification system has also been reported in Brazil due to incomplete and/or poorly filled information [35]. As a result, municipalities that have undetected cases in their territory are often incorrectly characterized as disease free zones in epidemiological reports. This fact can lead to a vicious cycle in which decreased surveillance leads to fewer detected cases which then leads to less disease prevention, control, and less surveillance.

Our statistical model demonstrated that municipalities that received technical training and support tended to have greater ability to detect cases. We also found that the detection probability decreases as distance to the notification center increases. The notification centers are those located in municipalities that are historically known for having the highest number of cases in their region, and hence their health professionals are more familiar with and prepared to detect the disease. Taken together, these results suggest that training professionals to better diagnose VL is critical to increase detection capacity. Nevertheless, we do note that our technical training and support results should be viewed with caution. Because the municipalities prioritized to receive technical training and support were selected because they were deemed to have high risk (i.e., moderate to high transmission of VL, including deaths), it is possible that the associations identified by our model is due to reverse causality (i.e., the higher number of cases, and thus higher detection probability, resulted in these counties receiving technical trainings and support). Determining if technical training and support did indeed improve detection will require more detailed data to allow for the assessment of how accurately health professionals diagnose VL patients before and after receiving this technical training and support.

### 4.3. Policy implications

Pernambuco was the first State of Brazil to create the Neglected Disease Control Program in 2011 (SANAR Program). This program has targeted the reduction or elimination of leishmaniasis and other diseases, such as tuberculosis, Hansen’s disease (leprosy), schistosomiasis, Chagas disease, filariasis, soil-transmitted helminthiases and trachoma [33]. Between 2015 and 2018, this program prioritized 11 municipalities (as shown in Fig 3) to finance prevention and control actions of VL through technical training and support. In the 2019–2022 quadrennium, the program planned to provide enhanced financial support to 19 municipalities, ten of which are new priority municipalities whereas 2 municipalities were removed from the priority list. The criterion for inclusion or exclusion of municipalities in the program is based on the number of cases and deaths in the period from 2013 to 2017.

We believe that these criteria to define priority municipalities could be complemented with our modeling results. For instance, a municipality with little agriculture and pasture and with technical training located close to a notification center is likely to be truly uninvaded if no cases have ever been detected. On the other hand, a municipality far from the notification center, with no technical training, and located in an area with a substantial amount of agriculture and pasture might have already been invaded, but the surveillance system might have routinely failed to detect VL cases due to low detection probability. In short, these results can help determine which municipalities would benefit from enhanced disease surveillance, mainly focusing on technical training and distribution of notification centers aimed at areas with the most need.

One of the main VLPCP strategies is based on early diagnosis and treatment of human cases [36], given that failure or late diagnosis directly compromises the patient’s prognosis [37–40]. Because early treatment considerably reduces VL mortality, regions with surveillance failures must be closely monitored and the causes of these failures properly identified and corrected.

Our modeling results reinforce the need for strategies such as technical training and support in both endemic areas for VL and areas at risk of having emergent cases (mostly surrounding other endemic municipalities). In particular, there is a need to better prepare the health teams of the municipalities that are most distant from the notification centers in order to make them self-sufficient and able to diagnose the cases that they are missing. If well directed, these interventions could bring real results on preventive vigilance, contributing to early diagnosis and, consequently, early treatment. Finally, although we have focused solely on Pernambuco state, it is likely that underreporting of VL cases is a common phenomenon across many Brazilian states. Therefore, analyses similar to the one presented in this study can potentially help Prevention and Control programs throughout Brazil.

To summarize, identifying possible municipalities with failure in notification can be an important foundation for optimizing skilled care and rapid treatment, in order to avoid serious cases and deaths. Critically, identifying risk factors associated with the disease helps to determine the probability of having or not cases in the local area. To have a major impact on reducing cases of VL, it is essential to understand and improve detection processes.
